# *Porphyromonas gingivalis* Fimbriae Induce Osteoclastogenesis via Toll-like Receptors in RAW264 Cells

**DOI:** 10.3390/ijms232315293

**Published:** 2022-12-04

**Authors:** Yuki Suzuki, Takeshi Kikuchi, Hisashi Goto, Yuhei Takayanagi, Shotaro Kawamura, Noritaka Sawada, Yoshikazu Naiki, Hisataka Kondo, Jun-ichiro Hayashi, Yoshiaki Hasegawa, Akio Mitani

**Affiliations:** 1 Department of Periodontology, School of Dentistry, Aichi Gakuin University, 2-11 Suemori-dori, Chikusa-ku, Nagoya 464-8651, Japan; 2 Department of Microbiology, School of Dentistry, Aichi Gakuin University, 1-100 Kusumoto-cho, Chikusa-ku, Nagoya 464-8650, Japan; 3 Department of Pharmacology, School of Dentistry, Aichi Gakuin University, 1-100 Kusumoto-cho, Chikusa-ku, Nagoya 464-8650, Japan

**Keywords:** osteoclasts, Mfa1 fimbriae, *Porphyromonas gingivalis*, bone loss, periodontal disease, toll-like receptors

## Abstract

The effect of Mfa1 fimbriae of *Porphyromonas gingivalis* on the progression of bone resorption remains unclear, especially compared with another fimbriae, FimA. We investigated the effect of Mfa1 on osteoclastogenesis together with FimA. We also investigated the role of Toll-like receptors (TLRs) in Mfa1 recognition during osteoclast differentiation. Receptor activator of nuclear factor κβ ligand (RANKL)-prestimulated RAW264 cells were used to examine the effects of purified Mfa1 fimbriae. The number of osteoclasts was examined by tartrate-resistant acid phosphate (TRAP) staining, osteoclast activation was investigated by bone resorption assays, and gene expression of differentiation markers was examined by quantitative real-time PCR. Transfection of Tlr2 and Tlr4 siRNAs into RAW264 cells was also employed and their role in Mfa1 recognition was investigated. Mfa1 effectively induced the formation of TRAP-positive multinucleated cells and activated osteoclasts. Mfa1 also increased gene expression of *Acp5*, *Mmp9*, and *Ctsk* in RANKL-prestimulated RAW264 cells compared with the control. The osteoclastogenesis induced by Mfa1 was significantly decreased in cells transfected with *Tlr2* or *Tlr4* siRNAs compared with control siRNA. Our results revealed the role of Mfa1 fimbriae in osteoclastogenesis that may contribute to the partial elucidation of the mechanisms of periodontal disease progression and the development of new therapeutic strategies.

## 1. Introduction

Periodontitis is a chronic inflammatory disease that is mainly caused by three species of bacteria called the Red complex (*Porphyromonas gingivalis*, *Tannerella forsythia*, and *Treponema denticola*) [1]. *P. gingivalis* is a gram-negative, biased anaerobic rod considered to be a keystone bacterium in the etiology of periodontal disease caused by multiple bacteria. In general, a significant infectious capacity is required for periodontopathic bacteria to destroy periodontal tissues, including attachment to periodontal tissue, invasion and disturbance of the host immune response, direct destruction, and escape from host immunity. The major virulence factors of *P. gingivalis* include fimbriae, lipopolysaccharide (LPS), and gingipain [2].

*P. gingivalis* has two types of fimbriae, long-type FimA fimbriae and short-type Mfa1 fimbriae, which are proteinaceous filamentous appendages [3]. They protrude from the bacterial cell surface and are thought to play important roles in attachment to the host tissue, biofilm formation, and coaggregation with streptococci and dendritic cells [4]. The *fimA* gene encodes the fimbrial major protein FimA, and the genotypes of *FimA* are I–V and Ib [5]. Genotype II is often detected in patients with severe periodontitis, whereas genotype I is typically detected in patients with mild periodontitis [5,6,7]. Mfa1 fimbriae consist of five proteins (Mfa1–5) with Mfa1 being the major subunit, which polymerizes on the fimbria axis [8].

FimA stimulates macrophages, gingival epithelial cells, and gingival fibroblasts to produce proinflammatory cytokines, such as interleukin (IL)-1, tumor necrosis factor (TNF)-α, and IL-6, which promote osteoclast differentiation and alveolar bone resorption [9,10,11]. *P. gingivalis* ATCC 33277 (wildtype) induces alveolar bone resorption in rats, and the *fimA*-deficient strain causes more bone resorption than the *mfa1*-deficient strain [12]. These findings suggest that Mfa1 has a more substantial effect on alveolar bone resorption than FimA. Additionally, *fimA* and *mfa1* double-deficient strains completely lose their ability to adhere to host cells. These results also suggest that both FimA and Mfa1 are important for *P. gingivalis* virulence.

Toll-like receptors (TLRs) on the cell surface recognize pathogen-associated molecular patterns. The host innate immune response to pathogens is mediated primarily by TLR signaling [13]. TLR2 and TLR4 are the most widely investigated extracellular innate immune receptors that recognize a variety of pathogen-associated molecular patterns and are closely related to the pathogenesis of periodontal disease [14]. In particular, TLR2 is important for *P. gingivalis* to produce proinflammatory cytokines [15,16]. It has been suggested that TLR2 and TLR4 may be involved in the recognition of *P. gingivalis* fimbriae [17,18], but a consensus has not been reached so far.

In the present study, we investigated the effects of *P. gingivalis* fimbriae, with particular attention to Mfa1 fimbriae, on the differentiation and activation of osteoclasts, which cause bone destruction of periodontal tissue compared with FimA fimbriae. Additionally, we examined the effects of TLR2 and TLR4 knockdown on osteoclast differentiation after fimbria stimulation.

## 2. Results

### 2.1. Mfa1 and FimA Fimbriae Promote RANKL-Mediated Osteoclast Differentiation

**Figure 1:**
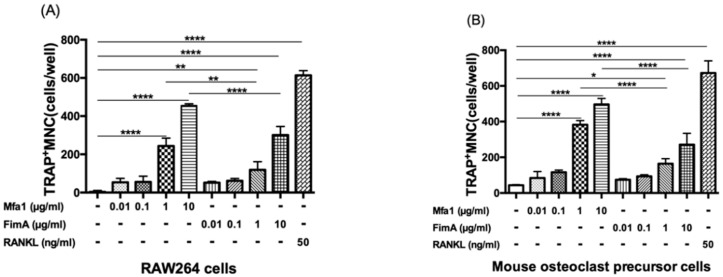
Effects of fimbriae on RANKL-mediated osteoclastogenesis. (**A**) RAW264 cells were prestimulated with 50 ng/mL RANKL for 24 h and then stimulated with 10 ng/mL, 100 ng/mL, 1 μg/mL, and 10 μg/mL Mfa1 and FimA fimbriae or 50 ng/mL RANKL every 48 h. After 96 h, the number of TRAP-positive multinucleated cells was counted. (**B**) Mouse bone marrow-derived osteoclast precursor cells were prestimulated with 50 ng/mL RANKL and 25 ng/mL M-CSF for 24 h and then stimulated with 10 ng/mL, 100 ng/mL, 1 μg/mL, and 10 μg/mL Mfa1 and FimA fimbriae or 50 ng/mL RANKL every 48 h. After 96 h, the number of TRAP-positive multinucleated cells was counted. Differences between groups were analyzed by ANOVA and Tukey’s test. Data are expressed as the mean ± SD (*n* = 3). * *p* < 0.05, ** *p* < 0.01, **** *p* < 0.0001.

RANKL-treated RAW264 cells and RANKL and M-CSF-treated mouse osteoclast precursor cells were stimulated with various concentrations of Mfa1 or FimA fimbriae to determine whether they affect RANKL-dependent osteoclastogenesis. Mfa1 and FimA stimulation of both cell types induced the formation of TRAP-positive multinucleated cells compared with controls in a dose-dependent manner (Figure 1A,B). Mfa1 had a significantly higher ability to induce osteoclast differentiation compared with 1 and 10 μg/mL FimA (Figure 1A,B).

### 2.2. Mfa1 and FimA Fimbriae Promote RANKL-Induced Osteoclastic Bone Resorption

**Figure 2:**
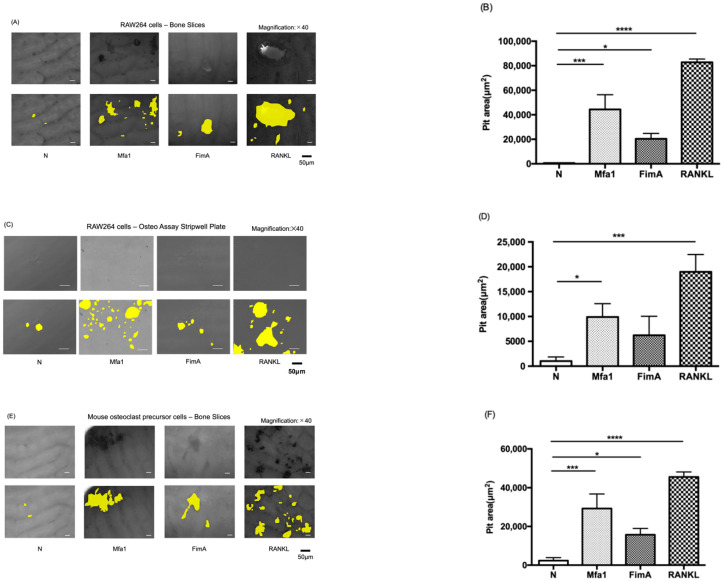
Effects of fimbriae on RANKL-mediated osteoclast activation. (**A**,**C**) RAW264 cells were prestimulated with 50 ng/mL RANKL for 24 h and then stimulated with 1 μg/mL Mfa1 and FimA fimbriae or 50 ng/mL RANKL every 48 h. After 120 h, images of the bone slice (**A**) and Osteo Assay Stripwell Plate (**C**) were obtained. Representative images are shown for each group. White and black bars indicate 50 µm widths. (**B**,**D**) Average area of pits on the bone slice (**B**) and Osteo Assay Stripwell Plate (**D**). Differences between groups were analyzed by ANOVA and Tukey’s test. Data are expressed as the mean ± SD (*n* = 3). * *p* < 0.05, *** *p* < 0.001, **** *p* < 0.0001. (**E**) Mouse bone marrow-derived osteoclast precursor cells were prestimulated with 50 ng/mL RANKL and 25 ng/mL M-CSF for 24 h and then stimulated with 1 μg/mL Mfa1 and FimA fimbriae or 50 ng/mL RANKL every 48 h. After 120 h, images of the bone slice were obtained. Representative images are shown for each group. White and black bars indicate 50 μm widths. (**F**) Average area of pits. Differences between groups were analyzed by ANOVA and Tukey’s test. Data are expressed as the mean ± SD (*n* = 3). * *p* < 0.05, *** *p* < 0.001, **** *p* < 0.0001.

To evaluate the effect of fimbriae on osteoclast activation, RAW264 cells were plated on bone slices and hydroxyapatite (HA) mineral surfaces and cultured for 120 h in the presence of Mfa1 or FimA fimbriae after RANKL prestimulation. The bone slice surface was partially resorbed by osteoclasts derived from RAW264 cells treated with RANKL and Mfa1 or FimA (Figure 2A). Interestingly, both Mfa1 and FimA significantly promoted bone pit formation compared with the control (Figure 2B). The osteoassay surface was also partially resorbed by osteoclasts derived from RAW264 cells treated with RANKL and Mfa1 or FimA (Figure 2C). Mfa1 significantly promoted HA pit formation compared with the control (Figure 2D). However, no significant pit formation areas were observed in the FimA group compared with the control (Figure 2D). Mouse osteoclast precursor cells were also plated on bone slices and cultured for 120 h in the presence of Mfa1 or FimA fimbriae after RANKL and M-CSF prestimulation. The bone slice surface was partially resorbed by osteoclasts derived from osteoclast precursor cells treated with RANKL and Mfa1 or FimA (Figure 2E). Interestingly, both Mfa1 and FimA significantly promoted bone pit formation compared with the control (Figure 2F).

### 2.3. Mfa1 Fimbriae Synergistically Induce Expression of RANKL-Dependent Osteoclast Differentiation Markers

**Figure 3:**
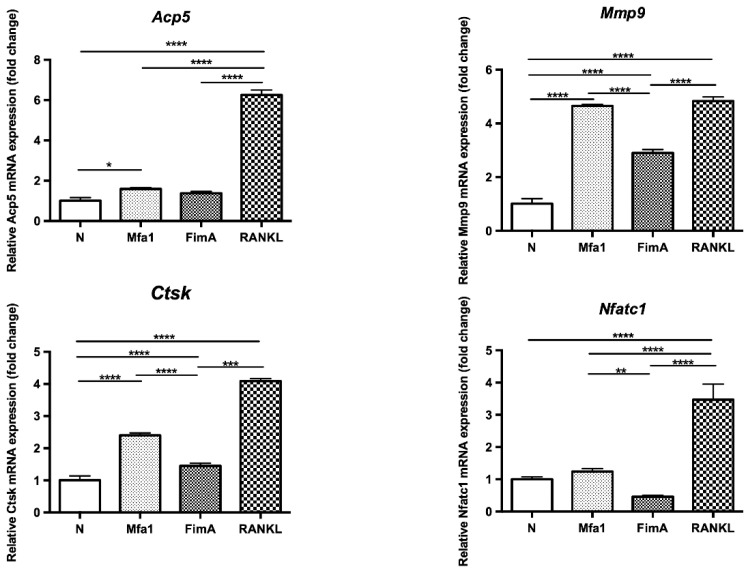
Effects of fimbriae on the expression of osteoclast differentiation marker genes in RANKL-prestimulated RAW264 cells. RAW264 cells were prestimulated with 50 ng/mL RANKL for 24 h and then cultured for 48 h in the presence of 1 μg/mL Mfa1 and FimA fimbriae or 50 ng/mL RANKL. mRNA levels were then examined by qPCR. Values are expressed as fold changes. Differences between groups were analyzed by ANOVA and Tukey’s test. Data represent the mean ± SD (*n* = 3). * *p* < 0.05, ** *p* < 0.01, *** *p* < 0.001, **** *p* < 0.0001.

RANKL-prestimulated RAW264 cells were stimulated with fimbriae or RANKL and then mRNA expression of TRAP (*Acp5*), matrix metalloproteinase 9 (*Mmp9*), cathepsin K (*Ctsk*), and nuclear factor of activated T cells c1 (*Nfatc1*) was examined by quantitative polymerase chain reaction (qPCR). Mfa1 significantly increased the expression of osteoclast differentiation markers *Acp5*, *Mmp9*, and *Ctsk* in RANKL-induced osteoclasts (Figure 3). However, both fimbriae (Mfa1 and FimA) did not affect the expression of *Nfatc1*, a master gene for osteoclast differentiation.

### 2.4. Mfa1 and FimA Fimbriae Increase Gene Expression of TLR2 and TLR4 in RANKL-Induced Osteoclasts

**Figure 4:**
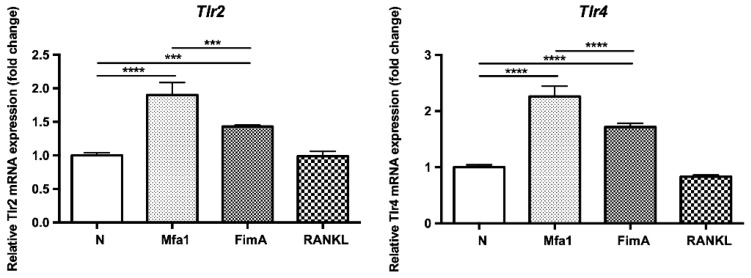
Mfa1 induces TLR2 and TLR4 gene expression in RANKL-prestimulated RAW264 cells. RAW264 cells were prestimulated with 50 ng/mL RANKL for 24 h and then cultured for 48 h in the presence of 1 μg/mL Mfa1 and FimA fimbriae or 50 ng/mL RANKL. Tlr2 and Tlr4 mRNA levels were determined by qPCR. Values are expressed as fold changes. Differences between groups were analyzed by ANOVA and Tukey’s test. Data represent the mean ± SD (*n* = 3). *** *p* < 0.001, **** *p* < 0.0001.

To examine whether fimbriae affect the expression of TLRs, RANKL-prestimulated RAW264 cells were stimulated with fimbriae or RANKL and then mRNA expression of TLR2 (*Tlr2*) and TLR4 (*Tlr4*) was investigated. As a result, Mfa1 and FimA significantly increased the expression of *Tlr2* and *Tlr4* in RANKL-induced osteoclasts (Figure 4).

### 2.5. Transfection of Tlr2 and Tlr4 siRNAs into RAW264 Cells

To determine whether TLRs on osteoclasts recognized Mfa1 and FimA, we examined the effects of silencing TLR2 and TLR4 expression. Tlr2 siRNA and Tlr4 siRNA-transfected RAW264 cells showed clear knockdown of *Tlr2* and *Tlr4* mRNAs, respectively, compared with control siRNA-transfected RAW264 cells (see Supplementary Figure S1A). Flow cytometric analysis also showed that surface expression of TLR2 and TLR4 was suppressed in siRNA-transfected RAW264 cells compared with control cells (see Supplementary Figure S1B).

### 2.6. Mfa1 Fimbriae Induce Osteoclast Differentiation Primarily through Recognition by TLR2

**Figure 5:**
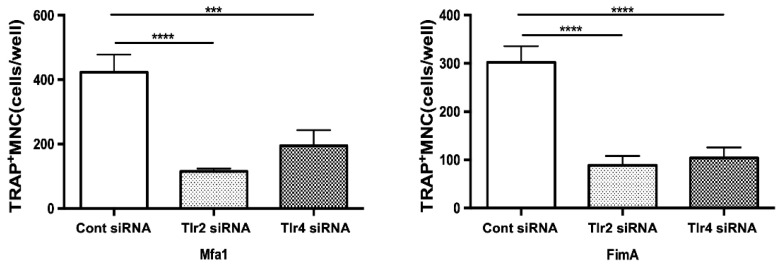
Suppression of TLR2 or TLR4 expression attenuates induction of osteoclastogenesis by Mfa1. Tlr2 siRNA and Tlr4 siRNA-transfected cells prestimulated with 50 ng/mL RANKL for 24 h were stimulated with 1 μg/mL Mfa1 and FimA fimbriae every 48 h. The number of TRAP-positive multinucleated cells was counted after 96 h. Differences between groups were analyzed by ANOVA and Tukey’s test. Data are expressed as the mean ± SD (*n* = 4). *** *p* < 0.001, **** *p* < 0.0001.

RANKL-prestimulated RAW264 cells with suppressed expression of TLR2 and TLR4 were stimulated by fimbriae and then osteoclast differentiation was examined by TRAP staining. Notably, among *Tlr2* siRNA-transfected cells stimulated by Mfa1, the number of TRAP-positive cells was markedly decreased compared with control siRNA-transfected cells (Figure 5). Additionally, suppression of *Tlr4* partially but significantly weakened the effect of Mfa1 on osteoclast differentiation (Figure 5). In the case of FimA stimulation, suppression of *Tlr2* and *Tlr4* resulted in significantly weakened osteoclast differentiation (Figure 5).

### 2.7. Mfa1 Fimbriae Induce Osteoclast Differentiation Markers Primarily through Recognition by TLRs

**Figure 6:**
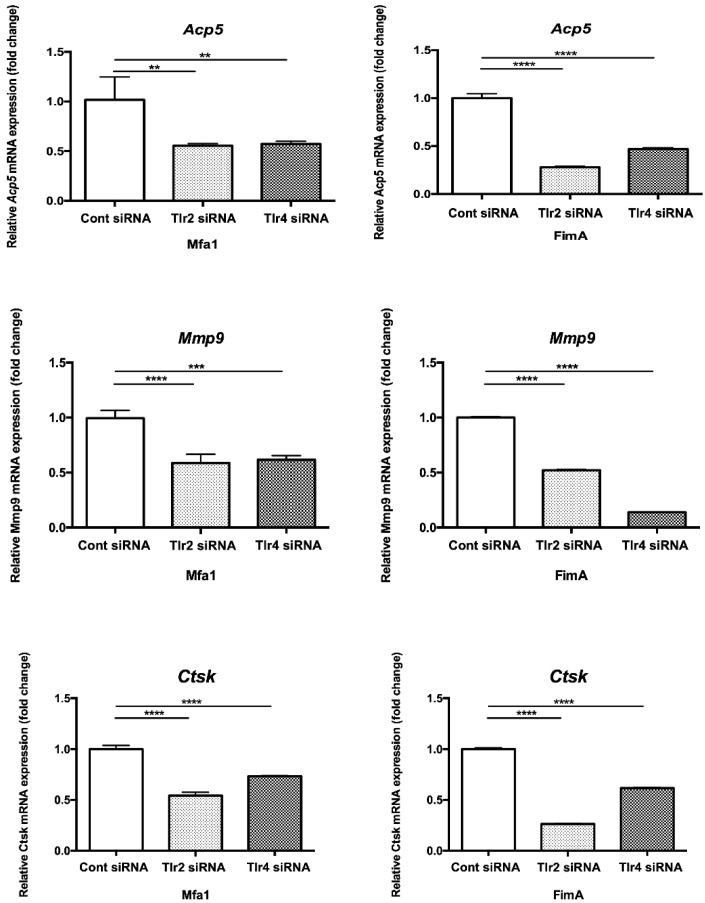
Suppression of TLR2 or TLR4 expression attenuates induction of osteoclast differentiation marker genes by Mfa1. Tlr2 siRNA and Tlr4 siRNA-transfected cells were prestimulated with RANKL for 24 h and then cultured for 48 h in the presence of 1 μg/mL Mfa1 or FimA fimbriae. mRNA levels were then examined by qPCR. Values are expressed as fold changes. Differences between groups were analyzed by ANOVA and Tukey’s test. Data represent the mean ± SD (*n* = 3). ** *p* < 0.01, *** *p* < 0.001, **** *p* < 0.0001.

RANKL-prestimulated RAW264 cells with suppressed expression of TLR2 and TLR4 were stimulated by fimbriae and then osteoclast differentiation marker expression was examined by qPCR. Expression of osteoclast differentiation markers *Acp5*, *Mmp9*, and *Ctsk* after Mfa1 stimulation was significantly decreased in *Tlr2* siRNA and *Tlr4* siRNA-transfected cells compared with control siRNA-transfected cells (Figure 6).

## 3. Discussion

In the present study, we demonstrated that *P. gingivalis* Mfa1 and FimA fimbriae promoted osteoclast differentiation and activation. Notably, the effects of Mfa1 on osteoclast differentiation appeared to be stronger than those of FimA. Hiramine et al. reported that the 67-kDa fimbriae (corresponding to Mfa1 fimbriae) of *P. gingivalis* induce osteoclast activation [19]. In their study, they used mouse primary bone marrow cells and stromal cell lines to confirm resorption pit formation in dentin slices under treatment with macrophage colony-stimulating factor (M-CSF), RANKL, dexamethasone, and 1α,25(OH)_2_ D_3_. Additionally, bone marrow cells and stromal cells were cocultured and many osteoclastogenesis-inducing factors were involved in their study. The findings suggested that Mfa1 fimbriae may promote osteoclastogenesis by stimulating receptors on stromal cells and increasing RANKL production. In the current study, we consider this indirect osteoclastogenesis to be a major difference because we examined direct effects on osteoclast progenitor cells. FimA fimbriae also stimulate bone resorption activity of calvarial bone cells on a bovine bone slice [20]. However, no direct effects of FimA on osteoclast differentiation or activation have been reported to our knowledge. Therefore, this is the first report on the direct effects of FimA and Mfa1 fimbriae on osteoclastogenesis. We observed no increase in Nfatc1 expression induced by fimbriae, which may be due to the stimulation time (48 h). In fact, we observed an increase in Nfatc1 expression at 8 and 24 h of stimulation with fimbriae (Supplementary Figure S2). In particular, the absorptive capacity of FimA-stimulated cells for minerals was not increased in a statistically significant manner unlike on bone slices. These data suggest that the ability of FimA to generate hydrogen ions via the ATP6i complex from osteoclasts is not as high as that of Mfa1. The differences in the inducibility of osteoclast differentiation and activation by various *P. gingivalis* fimbriae need to be investigated further. Recently, FimA and Mfa1 fimbriae of *P. gingivalis* were classified as novel V-type fimbriae [21], and their detailed structure, including trace components such as Mfa2-5, has been identified [4]. In studies investigating the relationship between bone resorption and *P. gingivalis* fimbriae, such fimbrial structures have not been considered. Future studies should focus on the detailed fimbrial structure to further explore the direct mechanism of osteoclastogenesis.

The proinflammatory mediator IL-1 has been suggested to directly promote osteoclast activation [22]. TNF-α has also been suggested to directly promote osteoclast differentiation [23]. LPS stimulates the survival and fusion of osteoclasts independently of RANKL, IL-1, and TNF-α [24]. LPS also induces various cytokines and mediators, such as IL-1, TNF-α, and prostaglandin E2, which play crucial roles in osteoclast differentiation and activation [25]. LPS appears to affect osteoclastogenesis in a complicated manner. Simultaneous stimulation by RANKL and LPS or staphylococcal lipoteichoic acid inhibits osteoclast differentiation induced by RANKL [26,27], whereas stimulation of osteoclast progenitors by LPS appears to promote osteoclastogenesis as well as osteoclast survival and activation [26,28,29]. Our preliminary results indicated that RANKL-unstimulated RAW264 cells, with a macrophage-like state, produced various proinflammatory cytokines upon the addition of Mfa1, and stimulation by Mfa1 alone did not differentiate macrophages into osteoclasts. Indeed, Hamada et al. reported that Mfa1 increases the expression of IL-1, IL-6, and TNF-α in mouse peritoneal macrophages [30]. Therefore, the effects of Mfa1 on osteoclast differentiation and activation in the present study were considered to occur in RANKL-induced osteoclast precursor cells. RANKL-primed macrophages promote osteoclastogenesis in a TNF-α-independent manner by *P. gingivalis* [31]. It has been reported that 41-kDa fimbriae of *P. gulae*, a periodontopathogenic bacterium in dogs, promote osteoclast differentiation upon costimulation with RANKL and 1α,25(OH)_2_D_3_ [32]. In actual bone resorption, many bone resorption-promoting factors would be produced in the surroundings, and the action of Mfa1 would be further enhanced.

TLRs recognize many bacterial components and play a decisive role in innate immunity [13]. Although *E. coli* LPS is recognized by TLR4 [13], *P. gingivalis* LPS is reported to be recognized by both TLR2 and TLR4 [33], suggesting that the biological response to the bacterial components of *P. gingivalis* is more complex. Recombinant FimA enhances inflammatory mediator production in human peripheral blood monocytic cells via TLR4 [34]. Moreover, FimA-like lipoproteins or lipopeptides related to FimA have been suggested to induce, at least in part, TLR2-mediated signaling and subsequent TNF-α production in macrophages [35]. In terms of Mfa1, the recognition receptor remains controversial. We recently reported that TLR4 is mainly important for the recognition of Mfa1 by gingival fibroblasts [17]. Takahashi et al. reported that TLR2 may be important for Mfa1 recognition by bronchial epithelial cells [18]. Our results suggested that TLR2, along with TLR4, may be important for the recognition of Mfa1 and FimA by osteoclast progenitor cells. Indeed, 67-kDa fimbriae of *P. gingivalis* induce osteoclast activation and its effect is attenuated by TLR2-neutralizing antibodies [19]. Future analysis using knockout mice or the CRISPR-Cas9 system should clarify the recognition receptors of Mfa1 and FimA.

This study has several limitations. First, because this was a cell-based in vitro study, future in vivo studies using infection model animals with an emphasis on analysis of alveolar bone resorption using a fimbria mutant strain of *P. gingivalis* should be conducted to clarify the role of Mfa1 in periodontitis. Second, osteoclasts are also induced to differentiate and activate by proinflammatory mediators derived from osteoblasts, macrophages, gingival fibroblasts, and epithelial cells. Therefore, it is necessary to study the effect of multiple cell types on the intercellular network of Mfa1. Third, this study did not examine the intracellular signaling of TLRs, the putative receptors of Mfa1. Such studies may determine which intracellular signaling pathways of TLRs regulate osteoclast differentiation and activation induced by Mfa1.

## 4. Materials and Methods

### 4.1. Cell Culture

The mouse macrophage cell line RAW264 was purchased from the RIKEN Cell Bank (Ibaraki, Japan). Mouse bone marrow-derived osteoclast precursor cells were purchased from COSMO BIO (Tokyo, Japan). Both cell types were cultured in α-minimum essential medium (MEM) (Thermo Fisher Scientific, Wilmington, DE, USA) containing 10% fetal bovine serum (MP Biomedicals, Santa Ana, CA, USA), 100 U/mL penicillin, and 100 µg/mL streptomycin in a 5% CO_2_ incubator at 37 °C.

### 4.2. Bacterial Strains and Growth Conditions

Mfa1 and FimA fimbriae were purified from *P. gingivalis* mutant JI-1 (*fimA* deleted) [36,37] and SMF-1 (*mfa1* deleted) [38,39] derived from ATCC 33277. JI-1 and SMF-1 cells were cultured under anaerobic conditions at 37 °C on Brucella HK agar medium (KYOKUTO, Tokyo, Japan) prepared by mixing 5% [volume/volume (*v*/*v*)] laked rabbit blood, 2.5 μg/mL hemin (Sigma-Aldrich, St. Louis, MO, USA), 5 μg/mL menadione (Sigma-Aldrich), and distilled water. Liquid medium was prepared by mixing trypticase soy broth (Thermo Fisher Scientific), 0.25% yeast extract (Thermo Fisher Scientific), and distilled water, followed by sterilization and the addition of 2.5 μg/mL hemin and 5 μg/mL menadione.

### 4.3. Purification of Fimbriae

Purification of Mfa1 fimbriae from JI-1 (*fimA* deleted) was performed using a standard protocol [36]. Briefly, *P. gingivalis* cells disrupted in a French pressure cell (OHTAKEWORKS, Osaka, Japan) were ultracentrifuged, and then the supernatant was precipitated with ammonium sulfate (50% saturation). The Mfa1 fimbrial fraction was separated by ion exchange chromatography (DEAE Sepharose Fast Flow chromatography, GE Healthcare Bio-Sciences AB, Uppsala, Sweden). The purity and identity of Mfa1 fimbriae were verified by sodium dodecyl sulfate-polyacrylamide gel electrophoresis and transmission electron microscopy. FimA fimbriae from SMF-1 (*mfa1* deleted) were purified in accordance with the protocol by Yoshimura et al. [40].

### 4.4. Tartrate-Resistant Acid Phosphatase (TRAP) Staining

RAW264 cells were prestimulated with 50 ng/mL receptor activator of nuclear factor κβ ligand (RANKL) (PeproTech, Rocky Hill, NJ, USA) for 24 h. Mouse bone marrow-derived osteoclast precursor cells were prestimulated with 50 ng/mL RANKL and 25 ng/mL macrophage colony-stimulating factor (M-CSF) (Sigma-Aldrich) for 24 h. Then, the cells were stimulated with 10 ng/mL, 100 ng/mL, 1 μg/mL, and 10 μg/mL Mfa1 and FimA fimbriae or 50 ng/mL RANKL every 48 h. After 96 h, the cells were washed with phosphate-buffered saline and fixed in 3.3% formaldehyde, a citric acid solution, and acetone for 5 min. The staining solution was prepared by mixing a Fastgarnet GBc BASE solution, sodium nitrite solution, Naphthol AS-BIPb, acetic acid solution, tartaric acid solution (all purchased from Sigma-Aldrich), and distilled water. After fixation, cells were washed with distilled water and stained in the solution for 30 min. After the cells were washed with distilled water and dried, TRAP-positive multinucleated cells containing three or more nuclei were counted under an optical microscope (BZ-X700, KEYENCE, Osaka, Japan).

### 4.5. Bone Resorption Assay

After washing a bone slice (BioVendor R&D, Brno, Czech Republic) and bone resorption activity evaluation plate (Osteo Assay Stripwell plate, Corning Lifesciences, Corning, NY, USA) with α-MEM, seeded RAW264 cells were prestimulated with 50 ng/mL RANKL for 24 h and mouse bone marrow-derived osteoclast precursor cells were prestimulated with 50 ng/mL RANKL and 25 ng/mL M-CSF for 24 h. Then, every 48 h, the cells were stimulated with 1 μg/mL Mfa1 and FimA fimbriae or 50 ng/mL RANKL. After 120 h, the cells were lysed in 5% sodium hypochlorite for 5 min. After washing with distilled water and drying, the absorption pit area was measured under the optical microscope. Three areas where pits had formed were randomly selected and the total area was measured using a BZ-X Analyzer (KEYENCE).

### 4.6. Real-Time qPCR

RAW264 cells were prestimulated with 50 ng/mL RANKL for 24 h. Then, the cells were stimulated with 1 μg/mL Mfa1 and FimA fimbriae or 50 ng/mL RANKL for 48 h. Total RNA was then extracted using NucleoSpin RNA (Macherey-Nagel Inc, Bethlehem, PA, USA) in accordance with the manufacturer’s protocol. The sample concentration was measured by a Thermo NANO DROP LITE (Thermo Fisher Scientific) and underwent cDNA conversion using a Biosystems GeneAmp PCR System (Thermo Fisher Scientific). Conditions were 37 °C for 15 min, 50 °C for 5 min, 98 °C for 5 min, and hold at 4 °C. Then, to quantify mRNA expression, real-time qPCR was performed using Taqman gene expression assays (Thermo Fisher Scientific) for mouse *Acp5* (Trap) (Mm00437135-m1), *Mmp9* (Mm00442991-m1), *Ctsk* (Mm00484039-m1), and *Nfatc1* (Mm00479445-m1) with TaqMan Universal PCR Master Mix (Thermo Fisher Scientific). mRNA levels were normalized to eukaryotic 18S rRNA (Hs99999901_s1). qPCR was performed using a StepOnePlus™ Real-Time System (Thermo Fisher Scientific). The thermocycling conditions were 40 cycles of 10 min at 95 °C, followed by 40 cycles of 15 sec at 95 °C and 1 min at 60 °C. Relative changes in gene expression were calculated using the 2^−ΔΔCt^ method. 18S rRNA (Hs99999901-s1) was used as an internal control.

### 4.7. siRNA Transfection

RAW264 cells at 60–80% confluence were transfected with siRNAs targeting *Tlr2* and *Tlr4* (Silencer Select Pre-designed siRNAs, Ambion, Austin, TX, USA) or non-targeting control siRNA using Lipofectamine RNAiMAX (Thermo Fisher Scientific) in Opti-MEM (Thermo Fisher Scientific). After 24 h, cells were collected, prestimulated with RANKL for 24 h, and then divided into two experimental systems. First, the cells were stimulated with 1 μg/mL Mfa1 and FimA fimbriae or RANKL for 48 h and then collected to analyze gene expression by qPCR. Second, cells were stimulated with 1 μg/mL Mfa1 and FimA fimbriae or 50 ng/mL RANKL every 48 h. After 96 h, the cells were stained with TRAP.

### 4.8. Flow Cytometry

siRNA-transfected RAW264 cells were stained with anti-mouse CD282 (TLR2) phycoerythrin (PE) (BioLegend, San Diego, CA, USA), anti-mouse CD284 (TLR4) PE (BioLegend), or isotype control PE (BioLegend) antibodies and then analyzed by flow cytometry using a MACSQuant analyzer (Miltenyi Biotec, Tokyo, Japan) and MACSQuantify software version 2.5 (Miltenyi Biotec).

### 4.9. Statistical Analysis

Statistical analysis was performed using PASW Statistics version 18.0 (SPSS Japan, Tokyo, Japan). Results were compared by analysis of variance (ANOVA) and Tukey’s multiple comparisons test. Comparisons between two independent groups were made using Student’s *t*-test. Data are expressed as the mean ± standard deviation (SD). Significant differences were accepted at less than 0.05.

## 5. Conclusions

Our findings demonstrate that Mfa1 fimbriae directly promote osteoclast differentiation and activation. Recognition of Mfa1 by TLRs on osteoclasts is important to facilitate osteoclastogenesis. Further studies focusing on the intracellular signaling of TLRs are necessary to reveal the underlying mechanism and develop therapeutic strategies for periodontal disease.

## Data Availability

Data are available on request.

## Supplementary Materials

The following supporting information can be downloaded at: https://www.mdpi.com/article/10.3390/ijms232315293/s1.
