# Supplementary material for: Porphyromonas gingivalis Fimbriae Induce Osteoclastogenesis via Toll-like Receptors in RAW264 Cells
Source: Int J Mol Sci. 2022 Dec 4;23(23):15293. doi: 10.3390/ijms232315293 (PMC9740495; doi:10.3390/ijms232315293)
Supplement: Supplementary file 1 [file ijms-23-15293-s001.zip › ijms-1948447-supplementary.pdf]

Supplementary Material

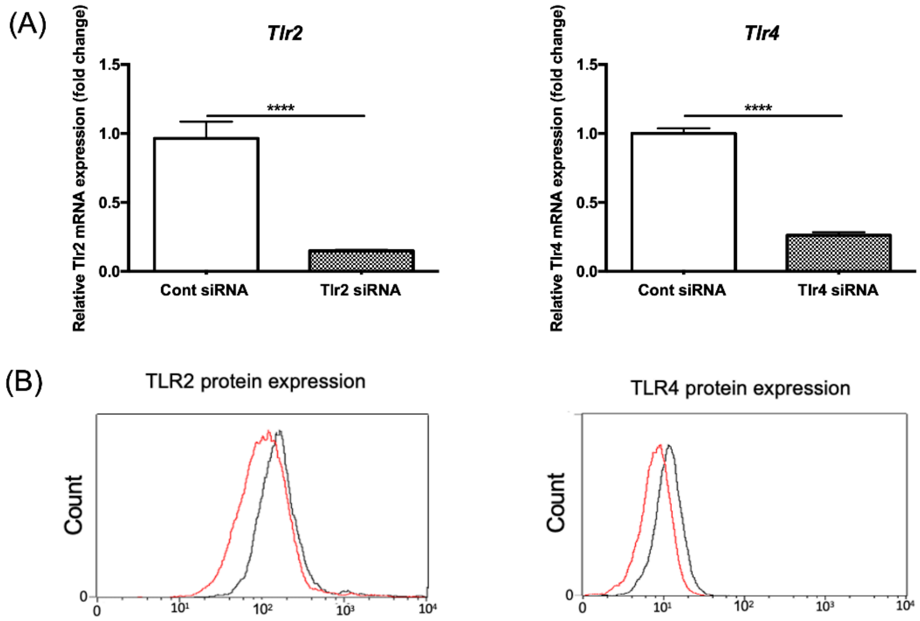

Supplemental Figure S1. Suppression of Tlr2 and Tlr4 in RAW264 cells by siRNAs. (A) Tlr2 and Tlr4 mRNA levels in siRNA-transfected RAW264 cells were examined by qPCR. Comparisons of two independent groups were performed using by Student's t-test. Data are expressed as the mean ± standard deviation (SD) (n=4). ****$p < 0.0001$. (B) Surface expression levels of TLR2 and TLR4 in siRNA-transfected RAW264 cells were examined by flow cytometry. RAW264 cells transfected with Tlr2 or Tlr4 siRNAs (red line) were compared with control siRNA-transfected RAW264 cells (black line).

Methods

Real-time Quantitative PCR

To quantify mRNA expression, real-time quantitative PCR (qPCR) was performed using Taqman gene expression assays (Thermo Fisher Scientific) for mouse Tlr2 (Mm00442346-m1) and Tlr4 (Mm00445273-m1) with TaqMan Universal PCR Master Mix (Thermo Fisher Scientific). mRNA levels were normalized to eukaryotic 18S rRNA (Hs99999901_s1). qPCR was performed using a Real-Time System (StepOnePlusTM Real-Time System, Thermo Fisher Scientific). The thermocycling conditions were 40 cycles of 10 min at 95°C, followed by 40 cycles of 15 sec at 95°C and 1 minute at 60°C. Relative changes in gene expression were calculated using the 2-ΔΔCt method. 18S rRNA (Hs99999901-s1) was used as an internal control.

Flow Cytometry

siRNA-transfected RAW264 cells were stained with anti-mouse CD282 (TLR2)

phycoerythrin (PE) (BioLegend, San Diego, CA, USA), anti-mouse CD284 (TLR4) PE (BioLegend), or isotype control antibody PE (BioLegend) and then analyzed by flow cytometry using an analyzer (MACSQuant analyzer, Miltenyi Biotec, Tokyo, Japan) and software (MACSQuantify software version 2.5, Miltenyi Biotec).

Legend

Supplemental Figure S1. Suppression of Tlr2 and Tlr4 in RAW264 cells by siRNAs. (A) Tlr2 and Tlr4 mRNA levels in siRNA-transfected RAW264 cells were examined by qPCR. Comparisons of two independent groups were performed using by Student's t-test. Data are expressed as the mean ± standard de-viation (SD) (n=4). ****p < 0.0001. (B) Surface expression levels of TLR2 and TLR4 in siR-NA-transfected RAW264 cells were examined by flow cytometry. RAW264 cells transfected with Tlr2 or Tlr4 siRNAs (red line) were compared with control siRNA-transfected RAW264 cells (black line).

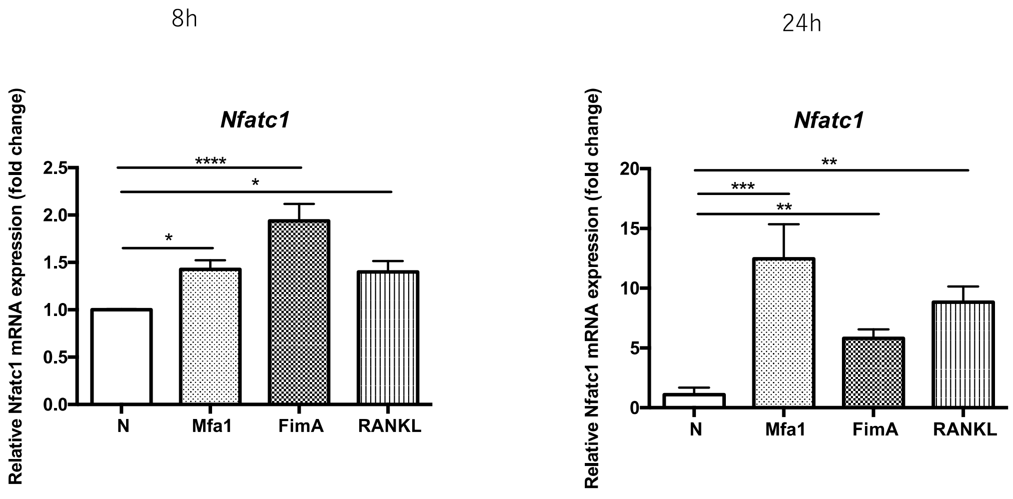

Supplemental Figure S2. Effects of fimbriae on expression of osteoclast differentiation marker genes in RANKL-prestimulated RAW264 cells. RAW264 cells were prestimulated with 50 ng/mL RANKL for 24 h and then cultured for 8 or 24 hours in the presence of 1 μg/mL Mfa1 and FimA fimbriae, or 50 ng/mL RANKL. mRNA levels were then examined by qPCR. Values are expressed as fold changes. Differences between groups were analyzed by ANOVA and Tukey's test. Data represent the mean ± SD (*n* = 3). *p < 0.05, **p < 0.01, ***p < 0.001, ****p < 0.0001.

Methods

Real-time Quantitative PCR

To quantify mRNA expression, real-time quantitative PCR (qPCR) was performed using Taqman gene expression assays (Thermo Fisher Scientific) for mouse *Nfatc1* (Mm00479445-m1) with TaqMan Universal PCR Master Mix (Thermo Fisher Scientific). mRNA levels were normalized to eukaryotic 18S rRNA (Hs99999901_s1). qPCR was performed using a Real-Time System (StepOnePlus™ Real-Time System, Thermo Fisher Scientific). The thermocycling conditions were 40 cycles of 10 min at 95°C, followed by 40 cycles of 15 sec at 95°C and 1 minute at 60°C. Relative changes in gene expression were calculated using the $2^{-\Delta\Delta Ct}$ method. 18S rRNA (Hs99999901-s1) was used as an internal control.

Legend

Supplemental Figure S2. Effects of fimbriae on expression of osteoclast differentiation marker genes in RANKL-prestimulated RAW264 cells. RAW264 cells were prestimulated with 50 ng/mL RANKL for 24 h and then cultured for 8 or 24 hours in the presence of 1 µg/mL Mfa1 and FimA fimbriae, or 50 ng/mL RANKL. mRNA levels were then examined by qPCR. Values are expressed as fold changes. Differences between groups were analyzed by ANOVA and Tukey's test. Data represent the mean ± SD (n = 3). $^*p < 0.05$, $^{**}p < 0.01$, $^{***}p < 0.001$, $^{****}p < 0.0001$.
